# Exploring the Safety and Satisfaction of Patients Injected With Collagen Biostimulators—A Prospective Investigation Into Injectable Poly‐l‐Lactic Acid (PLLA)

**DOI:** 10.1111/jocd.16723

**Published:** 2024-12-12

**Authors:** Bruna S. F. Bravo, Thamires Calvacante, Camila Silveira Nobre, Leonardo G. Bravo, Maria Carolina Zafra, Mariana Calomeni Elias

**Affiliations:** ^1^ Department of Dermatology Bravo Private Clinic Rio de Janeiro Brazil

**Keywords:** biostimulator, collagen, PLLA, poly‐l‐lactic acid, safety

## Abstract

**Background:**

Esthetic medicine has shifted significantly toward non‐surgical procedures, with the collagen biostimulator poly‐l‐lactic acid (PLLA) becoming increasingly popular for facial rejuvenation. However, the safety and patient satisfaction associated with PLLA facial treatments remain largely unexplored.

**Objective:**

This study aimed to evaluate the safety profile of PLLA treatment and assess patient mid‐ and long‐term satisfaction with the outcome.

**Methods:**

This prospective single‐center study was conducted at Bravo Clinic in Rio de Janeiro from August 2023 to August 2024. The patients' mid and lower face was treated by injecting Rennova Elleva (a collagen biostimulator containing 210 mg of PLLA) into the zygomatic and pre‐masseteric regions. All patients underwent monthly clinical evaluations for the first 150 days and kept diaries to self‐record any adverse effects during the first 30 days. Patient satisfaction was measured using the Global Aesthetic Improvement Scale (GAIS) on days 90 and 150 post‐injection.

**Results:**

The study included 52 female patients [mean age: 49.87 ± 10.7 years; mean body mass index (BMI): 23.99 ± 3.5 kg/m^2^]. Patients reported mild to moderate injection site reactions, including redness, pain, hardening, swelling, lumps, bruises, and skin discoloration, with most symptoms resolving within a few days. Clinically, no major adverse events were noted, except for one small, painless nodulation that resolved without intervention. On post‐injection Day 90, the average GAIS was rated at 2.32 ± 1.0. This rating remained stable at Day 150, with an average GAIS of 2.32 ± 0.8 (*p* = 0.862). Inverse correlations were identified between the duration of mild pain and GAIS (*r*_s_ = −0.546, *p* = 0.035), as well as between the duration of mild bruises and GAIS (*r*_s_ = −0.598, *p* = 0.019). The duration of moderate pain was significantly associated with age (*r*_s_ = −0.894, *p* = 0.041).

**Conclusion:**

Rennova Elleva, a PLLA‐based collagen biostimulator, is safe and well‐tolerated with a low incidence of adverse effects. It provides long‐lasting, satisfactory results for patients with mild to moderate facial sagging, rendering it an effective non‐surgical option for enhancing facial esthetics.

## 1 Introduction

Over the past decade, the field of esthetic medicine has witnessed a paradigm shift toward minimally invasive procedures that provide natural‐looking, long‐lasting results with little downtime. In this context, the innovation of collagen biostimulators has ushered in a new era of face rejuvenation treatments by offering patients a non‐surgical alternative to treating volume loss, skin laxity, and aging symptoms [1]. In contrast to conventional dermal fillers, which primarily address volume reduction by directly filling wrinkles, collagen biostimulators initiate a cascade of collagen synthesis that leads to gradual tissue regeneration and volume increase over time.

To date, there is a wide array of collagen biostimulators available, with poly‐l‐lactic acid (PLLA) being particularly popular. PLLA is a biocompatible synthetic polymer with broad biomedical applicability, including sutures and medical implants, due to its biodegradable and tissue‐friendly qualities. Specifically in esthetic medicine, PLLA is administered subcutaneously as an injectable suspension to increase the formation of collagen and gradually volumize the face. As the demand for PLLA grows—fueled by the desire for youthful, rejuvenated appearance—it is crucial to investigate both its safety and patients' satisfaction [2, 3, 4].

Adverse events related to PLLA injections are typically mild to moderate and transient in nature, including injection site reactions, swelling, bruising, and nodules. Although rare, major complications such as granuloma formation or tissue have been reported in a few cases, underscoring the importance of appropriate patient selection, injection technique, and post‐procedure monitoring. However, safety is not the only determinant of patient satisfaction, with esthetic outcomes and post‐treatment cosmesis also playing a central role [5].

PLLA biostimulation therapy warrants open communication, personalized treatment planning, and realistic goal setting, all of which are essential components of patient satisfaction. Importantly, PLLA biostimulators typically result in subtle yet significant improvements in skin quality and volume that evolve over time, requiring patience and realistic expectations. Conversely, this gradual nature of PLLA biostimulation therapy may also promote a sense of empowerment and control over the treatment process, as patients actively participate in their rejuvenation journey and may enjoy this progressive rejuvenation process without drastic changes to their appearance. Practitioners are, therefore, well advised to take the time to understand each patient's unique esthetic needs, preferences, and expectations, thus ensuring high patient satisfaction [6, 7, 8].

To date, prior research on safety and satisfaction in PLLA biostimulators is mainly derived from retrospective studies [9, 10, 11]. As a result, the external validity remains limited with potential biases. Therefore, we herein aim to prospectively assess the risk profile of PLLA treatment while seeking to understand patient satisfaction with PLLA biostimulation therapy. Our insights on safety and patient satisfaction in PLLA treatment can help inform patients on what to expect post‐injection and may advance the field of esthetic medicine toward safer, more effective, and satisfying solutions for facial rejuvenation and enhancement.

## 2 Materials and Methods

### 2.1 Study Setup

This prospective single‐center study aimed to investigate the safety and satisfaction following a single‐session treatment on the face with the cosmetic filler Rennova Elleva. The study was conducted between August 2023 and August 2024 in the private practice of the senior author, Clínica Bravo in Rio de Janeiro, Brazil. Ethical approval was. This study was performed in adherence to the Declaration of Helsinki (1996) and in accordance with regional laws and good clinical practice.

### 2.2 Inclusion and Exclusion Criteria

We applied strict inclusion and exclusion criteria to ensure the safety of all patients as well as the integrity and validity of the results. The exclusion criteria were clearly defined to avoid potential confounding factors that could impact the study's outcomes. Individuals under the age of 18 were excluded to maintain an adult population, while no pregnant or breastfeeding women were enrolled due to potential risks to both mother and child. Similarly, participants with a history of autoimmune diseases were not eligible to prevent complications that could arise from immune system interactions. Those using immunosuppressants or anti‐inflammatory medications were not included, as these drugs could potentially interfere with the study's treatments and outcomes. In addition, individuals with permanent facial implants, such as polymethyl methacrylate (PMMA) or silicone, were excluded due to the potential for these materials to affect the treatment results or cause adverse reactions. Participants who had received temporary or semi‐permanent skin fillers in the face within the last 12 months were also not eligible to ensure that recent cosmetic procedures did not skew the study's findings. Additionally, patients using topical or oral products that could significantly affect their skin condition were excluded from the study. Informed consent was obtained from all patients involved.

### 2.3 Product Specifications

Rennova Elleva contains 210 mg of PLLA, 132 mg of carboxymethylcellulose, and 178 mg of mannitol apirogenic. It is prepared in a sterile lyophilized form for deep dermal, subcutaneous, or supraperiosteal injection. The preparation consisted of 12 mL of sterile water for injection mixed with 2 mL of lidocaine without a vasoconstrictor. The Rennova Elleva vial was then placed in a Rennova Mixer 2.0, a professional mixing device, where it was shaken horizontally at 2000 oscillations per minute for 1 min to ensure optimal homogenization. From this solution, 8 mL (containing 120 mg of PLLA at a concentration of 15 mg/mL) was applied to the mid and lower thirds of the face.

### 2.4 Procedure

The procedure began with cleansing the skin using a 0.5% alcoholic chlorhexidine solution, followed by the application of a 4% lidocaine‐based anesthetic cream for 30 min. After removing the cream, a 22G 50 mm cannula (Rennova, Goiânia, Brazil) was used for the injections. An entry point was created with a 21G needle at a 30°–45° angle to the skin surface. For the treatment of the mid and lower face, the entry points were as follows:

1. Zygomatic region: The entry point was in the subcutaneous layer at the intersection of a horizontal line drawn from the tragus to the subnasale and a vertical line over the lateral orbital rim.
2. Pre‐masseteric region: The entry point was in the subcutaneous layer of the lower pre‐masseteric region, immediately above the mandibular body, at the anterior border of the masseter muscle.

A linear retro‐injection technique without bolus was used for all treatments. All injections were administered by the same experienced dermatologist. Immediately post‐injection, a massage with a 2% chlorhexidine degerming solution was performed to distribute the product evenly. Patients were instructed to continue this massage for 5 days, five times a day for 5 min each session. A sterile occlusive dressing was applied at each entry point.

### 2.5 Global Aesthetic Improvement Scale (GAIS) Assessment

The GAIS was used to evaluate patient satisfaction at Day 90 and Day 150. GAIS is a five‐point scale that evaluates the overall esthetic improvement in appearance compared to pre‐treatment, with ratings ranging from exceptional improvement (1) and very improved (2) through improved (3) to unaltered (4) and worsened (5). Rather than assessing individual components, the entirety of the esthetic improvement is considered. Based on the injection locations targeted in this study, the GAIS naturally focused on the overall esthetic improvement of the mid‐ and lower face.

### 2.6 Patient Diary

Patients received a diary to subjectively record any adverse effects during the first 30 days post‐procedure. Patients were instructed to indicate specific symptoms/signs (“redness”, “pain”, “hardening”, “swelling”, “lumps”, “bruise”, and “skin discoloration”) and the intensity (mild, moderate, or severe) of these signs/symptoms. The diaries were collected and analyzed at the day 30 visit.

### 2.7 Clinical Evaluation

Each patient underwent six monthly clinical evaluations on Days 0, 30, 60, 90, 120, and 150. During each of these evaluations, a comprehensive anamnesis and physical examination, including facial skin palpation, were conducted to assess for any adverse events.

### 2.8 Statistical Analysis

Differences between follow‐up periods within the investigated groups were determined using paired samples t‐test. Bivariate correlation analysis between the investigated parameters was performed employing Spearman's correlation coefficient (*r*_s_). Statistics analysis were performed using SPSS Statistics 27 (IBM, Armonk, NY, USA), and differences were considered statistically significant at a probability level of ≤ 0.05 to guide conclusions. Results are presented as mean values and their respective 1× standard deviation with mean (SD), unless indicated differently.

## 3 Results

### 3.1 Demographics

Our prospective study included 52 female patients with an average age of 49.87 years (SD: 10.7; range: 33–74 years). The patients' average body mass index (BMI) was 23.99 kg/m^2^ (SD: 3.5) at the start of the study (day 0), which increased slightly to 24.67 kg/m^2^ (SD: 4.0) by day 150 (*p* = 0.261).

### 3.2 GAIS Assessment

GAIS data were collected from all (*n* = 52, 100%) patients on Days 90 and 150. At Day 90, the average GAIS was 2.32 (SD: 1.0), indicating that patients generally perceived their esthetic improvement as “improved”. This rating remained stable at Day 150, with an average GAIS of 2.32 (SD: 0.8) (*p* = 0.862).

### 3.3 Patient Diary

The patient diary was completed by 69.2% (*n* = 36) of patients. Patients reported a range of mild to moderate injection site reactions. Redness at the injection site was reported in 8 cases (22.2%), with 7 cases being mild and 1 case being moderate. The average duration of redness was 1.71 days (SD: 0.8) for mild cases and 1 day for the moderate case. Pain at the injection site was reported in 28 cases (77.8%), with 23 cases being mild and 5 cases being moderate. The average duration of pain was 3.13 days (SD: 1.6) for mild cases and 2.00 days (SD: 0.7) for moderate cases. Hardening at the injection site was reported in 7 cases (19.4%), with all cases being mild and an average duration of 1.71 days (SD: 1.1). Swelling at the injection site was reported in 14 cases (38.9%), with 13 cases being mild and 1 case being moderate. The average duration of swelling was 2.50 days (SD: 1.0) for mild cases and 3 days for the moderate case. Lump formation at the injection site was reported in 3 cases (8.3%), with all cases being mild. The average duration of lump formation was 3.67 days (SD: 2.5). Bruises at the injection site were reported in 20 cases (55.6%), with 19 cases being mild and 1 case being moderate. The average duration was 3.50 days (SD: 1.8) for mild cases and 1 day for the moderate case. Skin discoloration at the injection site was reported in 7 cases (19.4%), with all cases being mild and an average duration of 2.14 days (SD: 1.1).

Inverse correlations were found between the duration of mild pain and GAIS (*r*_s_ = −0.546, *p* = 0.035), as well as between the duration of mild bruises and GAIS (*r*_s_ = −0.598, *p* = 0.019). Additionally, the duration of moderate pain was strongly associated with age (*r*_s_ = −0.894, *p* = 0.041) (Table 1).

**TABLE 1:** Summary of Spearman's correlation coefficients (*r*_s_) calculated for the parameters investigated in this study.

	Age	BMI (D0)	BMI (D150)	GAIS (D90)	GAIS (D150)
Age					
Correlation coefficient	1.000	0.205	0.235	0.264	0.202
Sig. (2‐tailed)		0.229	0.258	0.151	0.224
BMI (D0)					
Correlation coefficient	0.205	1.000	0.944**	−0.263	0.084
Sig. (2‐tailed)	0.229		**< 0.001**	0.152	0.682
BMI (D150)					
Correlation coefficient	0.235	0.944**	1.000	−0.129	0.131
Sig. (2‐tailed)	0.258	**< 0.001**		0.539	0.532
GAIS (D90)					
Correlation coefficient	0.264	−0.263	−0.129	1.000	0.185
Sig. (2‐tailed)	0.151	0.152	0.539		0.366
GAIS (D150)					
Correlation coefficient	0.202	0.084	0.131	0.185	1.000
Sig. (2‐tailed)	0.224	0.682	0.532	0.366	
Redness (Mild)					
Correlation coefficient	−0.136	−0.386	−0.053	0.335	−1.000**
Sig. (2‐tailed)	0.771	0.393	0.933	0.516	
Pain (Mild)					
Correlation coefficient	−0.101	0.157	−0.049	−0.060	−0.546*
Sig. (2‐tailed)	0.646	0.474	0.862	0.809	**0.035**
Pain (Moderate)					
Correlation coefficient	−0.894*	−0.224	−0.632	−0.500	
Sig. (2‐tailed)	**0.041**	0.718	0.368	0.391	
Hardening (Mild)					
Correlation coefficient	−0.090	0.299	0.632	−0.474	0.500
Sig. (2‐tailed)	0.847	0.515	0.368	0.282	0.500
Swelling (Mild)					
Correlation coefficient	−0.221	0.075	−0.295	−0.483	−0.015
Sig. (2‐tailed)	0.447	0.799	0.408	0.095	0.968
Lumps (Mild)					
Correlation coefficient	1.000**	0.500		−1.000**	
Sig. (2‐tailed)		0.667			
Bruises (Mild)					
Correlation coefficient	−0.245	−0.337	−0.382	−0.598*	−0.427
Sig. (2‐tailed)	0.312	0.158	0.198	**0.019**	0.146
Skin discoloration (Mild)					
Correlation coefficient	0.711	0.543	0.866	0.544	−0.866
Sig. (2‐tailed)	0.073	0.208	0.333	0.456	0.333

*Note:* Single moderate cases have been excluded due to the inability to compute correlation coefficients. Statistically significant values are highlighted in bold.

* Indicate statistical significance, with * representing *p* < 0.05.

** Representing *p* < 0.01.

### 3.4 Clinical Evaluation

All (*n* = 52, 100%) patients were clinically evaluated on Days 0, 30, 60, 90, 120, and 150. In the clinical evaluations, no major adverse events were reported except for one patient. At Day 60, a small (4 mm), painless, non‐visible nodulation was detected at the right pre‐zygomatic area during a physical examination, which resolved completely by Day 90 without intervention. Three patients reported minimal palpable lumps within the first few days, all of which disappeared within a week.

## 4 Discussion

According to the International Society of Aesthetic Plastic Surgery (ISAPS), 18.8 million non‐surgical procedures were performed worldwide in 2022, marking a significant increase of 57.8% within 4 years [12]. This upward trajectory reflects a paradigm shift in esthetic medicine toward less invasive methods. Although exact numbers for yearly PLLA procedures remain unspecified, the overall market trends underscore its growing relevance. As reported by Markets and Markets in 2023, the global PLLA market was valued at 1.5 billion USD, with projections estimating that it will reach 3.3 billion USD by 2028 [13]. In addition, Ianhez et al. recently reviewed complications associated with collagen biostimulators in 55 cases across Brazil [14]. They identified nodule formation, edema, and bacterial infection as potential adverse events, with the face being the most commonly affected area. Although these complications are rare, their occurrence, coupled with the growing demand for PLLA, highlights the need for more comprehensive studies to evaluate safety and patient satisfaction. Therefore, we herein prospectively evaluated both clinical and patient‐centered outcomes after facial PLLA biostimulation treatment with a single‐session injection of Rennova Elleva.

Importantly, in the context of facial esthetic treatments, modern outcome measurements should encompass both clinical indicators and patient perspectives [15]. Traditional mono‐perspective clinical assessments alone may not fully capture the subjective experiences of patients, which are crucial for determining the true success of esthetic interventions [16, 17]. Therefore, integrating patient‐reported outcomes provides a more holistic view of treatment efficacy and patient satisfaction. Our study leveraged self‐reported patient diaries to collect detailed post‐treatment experiences, providing a thorough view of patient‐reported side effects. Redness and pain at the injection site were the most commonly reported symptoms, documented in 22.2% and 77.8% of cases, respectively. These symptoms were generally mild and short‐lived, indicative of a typical response to the treatment. While minor symptoms such as hardening, swelling, lump formation, bruises, and skin discoloration were also noted, the majority were minimal and transient. It is worth mentioning that the relatively high prevalence of these minor adverse effects is likely inflated due to the study's methodology, whereby patients were hyper‐aware and meticulously recorded any changes in their condition. This heightened sensitivity and attentiveness among patients might lead to over‐reporting, and thus, these figures should be interpreted with caution [18]. The discrepancy between the incidence of patient‐reported symptoms and clinical expert evaluations further corroborates this point. In fact, clinically, only one minor adverse event was recorded, namely, a small, painless nodulation that resolved without intervention. This stark contrast emphasizes the need to cross‐check patient‐reported data with clinical assessments to obtain an accurate picture of PLLA's safety and efficacy.

Particularly in the field of esthetic medicine, the success of treatment should not be exclusively based on procedural safety. Instead, patient satisfaction with the esthetic result must also be taken into account. In this study, patients were asked to rate how satisfied they were with their appearance following treatment with PLLA on the 5‐point GAIS from 1 (“exceptional improvement”) to 5 (“worsened”). We found that at Day 90, the average GAIS was rated at 2.32 which remained stable at an identical average GAIS of 2.32 60 days later. This high‐level stability in GAIS scores over time indicates consistently high patient satisfaction and the lasting efficacy of PLLA treatments. The results presented herein are comparable with the previous literature in the field of facial esthetics, underlining the effectiveness of the products used in this study. For example, Dai et al. reported mean GAIS values of 2.11 and 2.16 12 weeks after injection of Princess VOLUME and Restylane, both hyaluronic acid (HA) dermal fillers, in the correction of moderate‐to‐severe nasolabial fold, respectively [19]. Similarly, in a recent meta‐analysis of randomized clinical trials on patients after administering HA, collagen, PLLA, and autologous fat implantation, Stefura et al. reported pooled GAIS scores of 2.21 and 2.32 after 1 and 6 months, respectively [20]. It is important to note that they also revealed a decrease in GAIS scores between months 6 and 12, which is beyond the evaluation period of our study. Therefore, further studies are needed to assess the long‐term durability and effectiveness of PLLA beyond 150 days.

Our analyses revealed inverse correlations between the duration of mild pain and GAIS scores, as well as between the duration of mild bruises and GAIS scores. Interestingly, while mild pain did not impact GAIS scores at Day 90, this association became statistically significant at Day 150 (Table 1). The lack of a significant correlation between the duration of mild pain and GAIS score at Day 90 may reflect patients' anticipation of some post‐procedural discomfort, making them more lenient in their evaluation at the 3‐month mark. In other words, patients could have accepted the pain as an expected part of the recovery process after the procedure. By Day 150, however, when patients have had the opportunity to reflect more comprehensively on their entire post‐treatment experience, they may have perceived the pain more negatively in retrospect, contributing to the significant correlation with the GAIS score at this later stage. However, further investigation into these time‐dependent correlations is necessary. Overall, our findings underscore the critical role of effective pain management in enhancing patient satisfaction. Pain, even when mild, can significantly affect patient's perception of the treatment's success. Therefore, comprehensive pain management can lead to overall improved outcomes. This aspect is particularly relevant in the realm of esthetic treatment, with patient satisfaction being directly linked to the comfort and experience during the recovery process. Pain, if not adequately managed, may overshadow the positive esthetic results achieved through the treatment. In this context, it is also important to note that the duration of moderate pain was significantly associated with age. This correlation implies that older patients may experience and report pain differently compared to younger individuals. The increased perceptiveness and reactivity to pain among older patients could be attributed to various physiological and psychological factors. For instance, older adults often have thinner skin and reduced subcutaneous fat, which might make them more susceptible to pain and bruising. Additionally, age‐related changes in pain perception and threshold could contribute to this higher sensitivity [21, 22, 23]. Understanding this age‐related difference in pain perception is crucial for tailoring treatment plans and post‐procedure care: for older patients seeking PLLA facial injections, it is beneficial to implement targeted pain relief strategies, thereby ensuring high satisfaction levels after collagen biostimulator treatments. This might also include pre‐treatment counseling to set realistic expectations, the application of topical anesthetics during the procedure, a more proactive use of analgesics, and the preparation of a robust post‐treatment care plan. Further, follow‐up appointments could be tailored to monitor and address any ongoing discomfort promptly.

**FIGURE 1:**
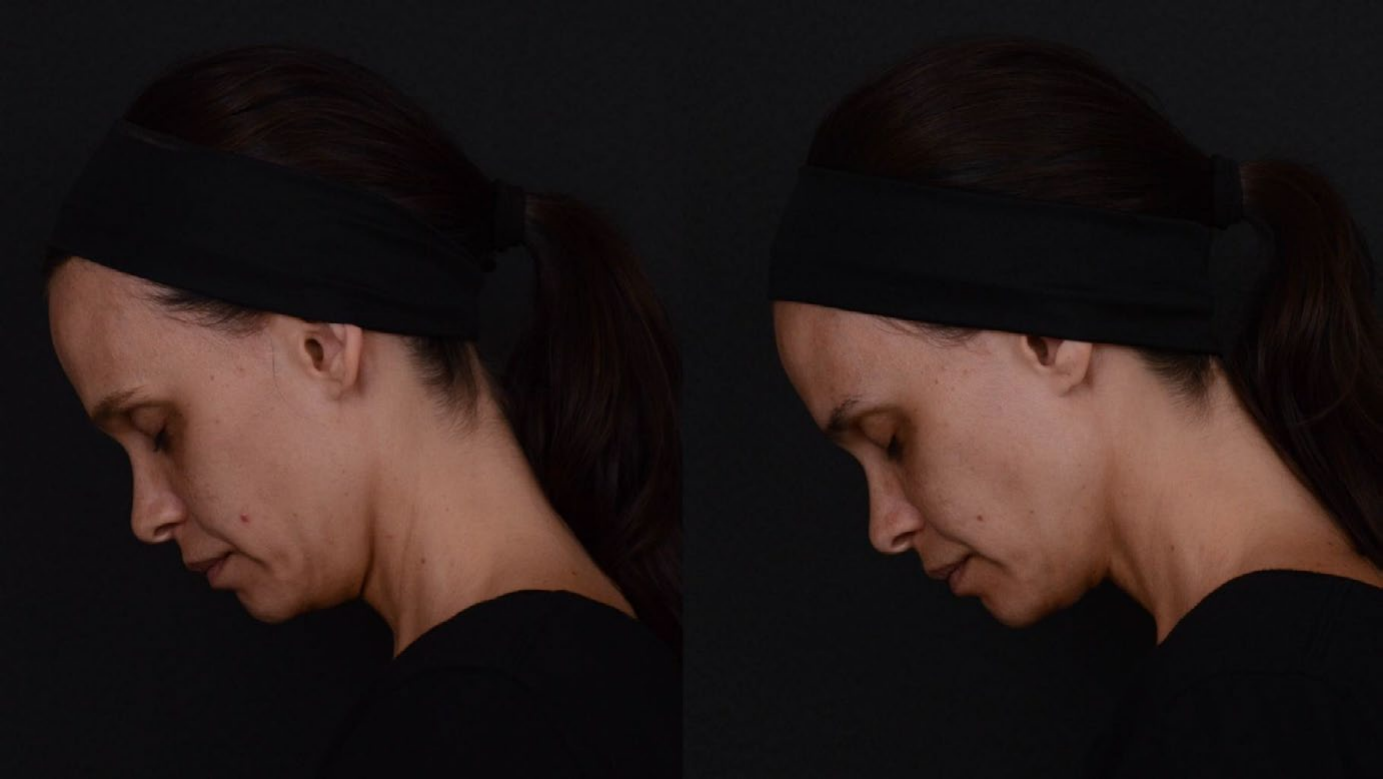
Side view comparison of mid and lower face appearance before (left) and 30 days after (right) treatment with Rennova Elleva, a PLLA‐based collagen biostimulator. Injections were targeted at the zygomatic and pre‐masseteric regions (see Figure 4 for details). It is worth noting that the images were taken with the head tilted downward to better illustrate the areas of the face most affected by the treatment. However, this is not a typical view used in cosmetic evaluations, which could potentially lead to a misinterpretation of the esthetic results. This limitation should be taken into account when reviewing the images.

**FIGURE 2:**
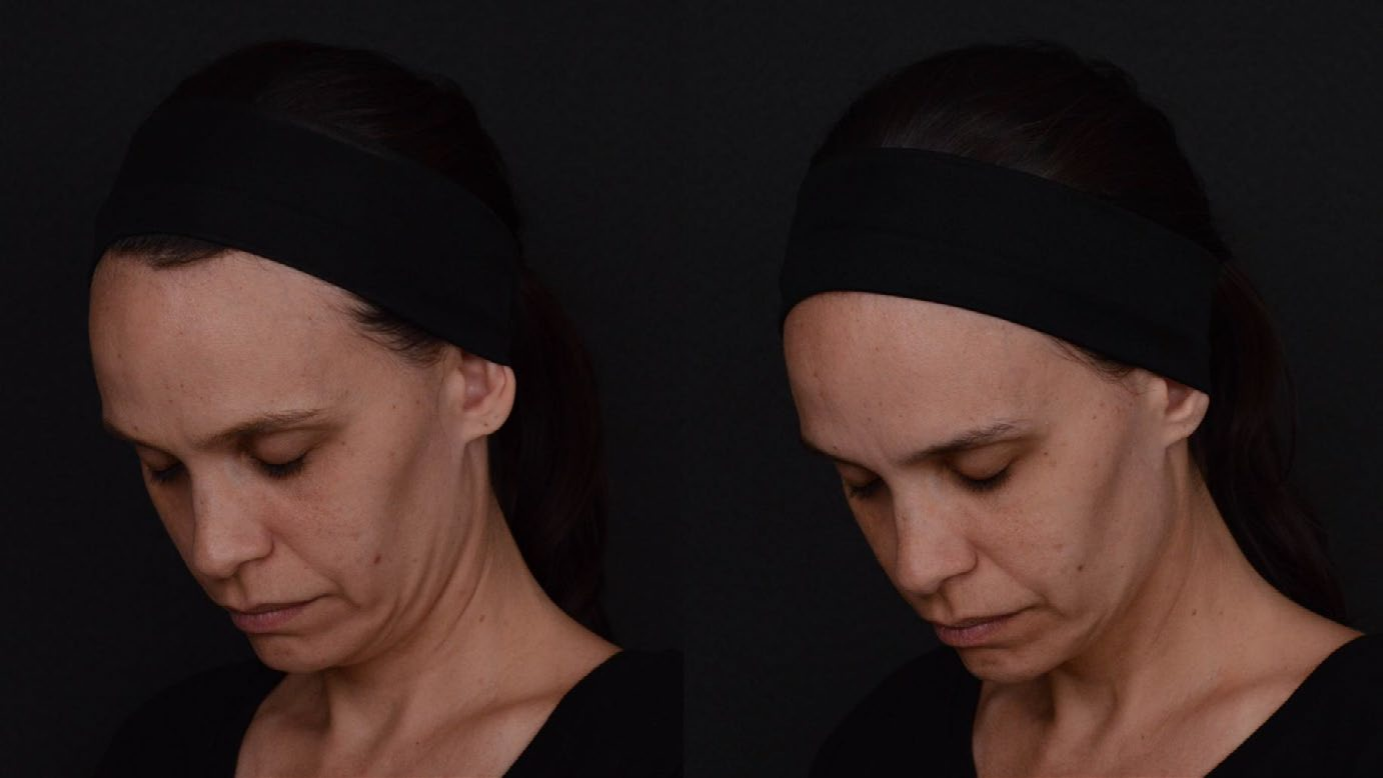
Oblique view comparison of mid and lower face appearance before (left) and 30 days after (right) treatment with Rennova Elleva, a PLLA‐based collagen biostimulator. Injections were targeted at the zygomatic and pre‐masseteric regions (see Figure 4 for details).

**FIGURE 3:**
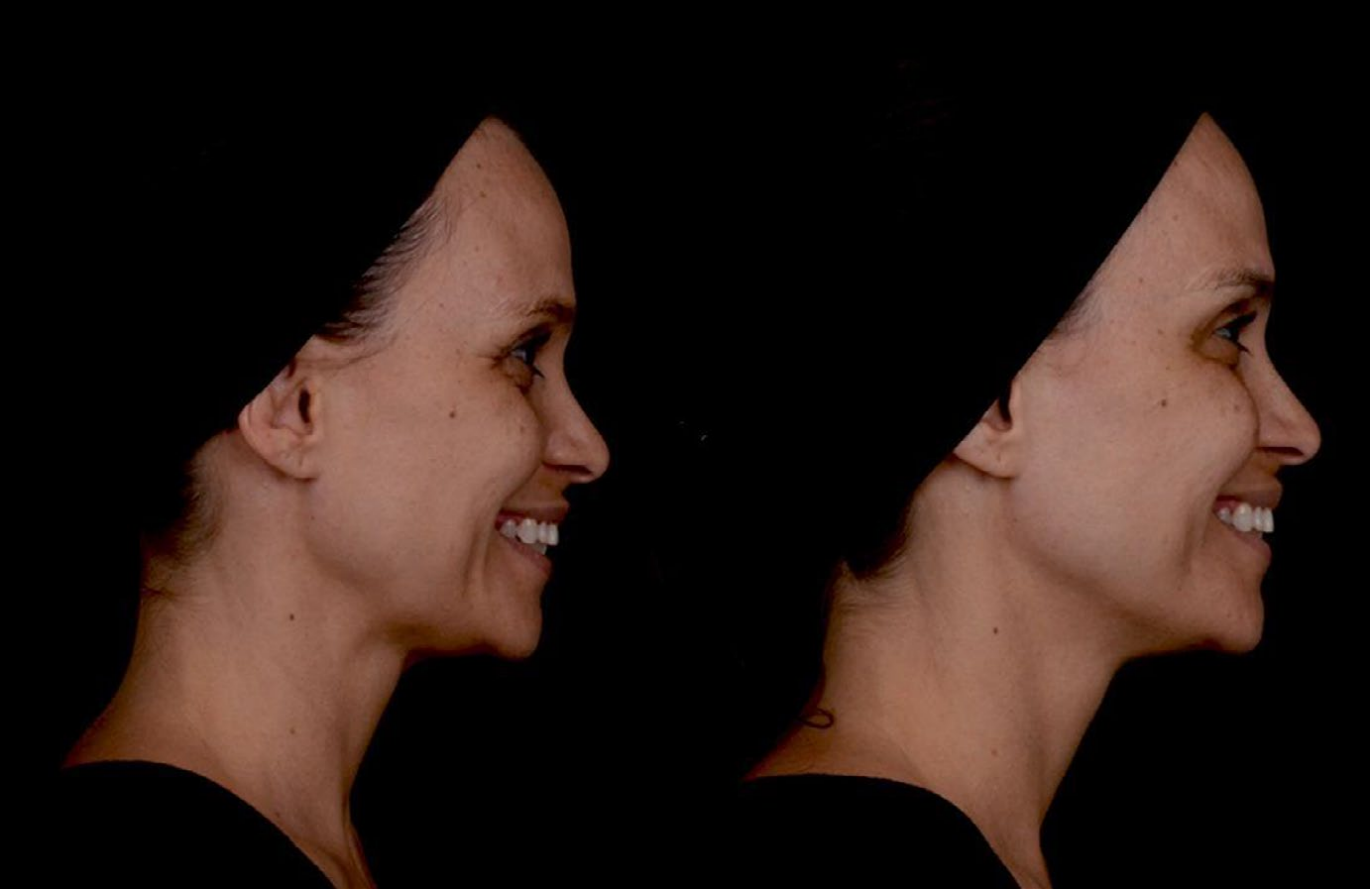
Side view comparison of mid and lower face appearance before (left) and 150 days after (right) treatment with Rennova Elleva, a PLLA‐based collagen biostimulator. Injections were targeted at the zygomatic and pre‐masseteric regions (see Figure 4 for details).

In conclusion, our study demonstrates that the collagen biostimulator PLLA is a safe and effective option for non‐surgical facial esthetic treatments, offering cosmetic improvements with high patient satisfaction and minimal adverse effects (Figures 1, 2, 3). The findings suggest that while patients may report a series of minor symptoms post‐treatment, these are generally short‐lived and do not significantly detract from the overall positive outcomes. The stability of GAIS ratings over time further emphasizes the long‐lasting satisfactory benefits of Rennova Elleva. Practitioners should, however, be sensitized to our finding that elderly patients may be particularly susceptible to pain perception, which may compromise their satisfaction with the outcome. For these pain‐sensitive patients, effective analgetic management strategies can help ensure that the positive esthetic outcomes are not marred by discomfort.

**FIGURE 4:**
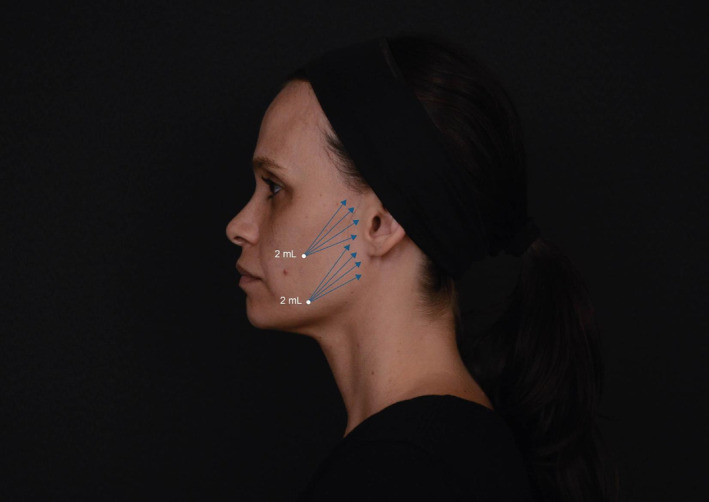
Side view illustrating the entry points and injection patterns for the administration of Rennova Elleva as a PLLA‐based collagen biostimulator.

## 5 Limitations

The study's findings ought to be interpreted in light of its inherent limitations. One significant limitation is the subjective nature of the data collection. As previously mentioned, by relying on patients to self‐report and document all changes they notice, there is a risk of exaggeration or misinterpretation of side effects, which might make them seem more concerning than they are in reality. However, it is also important to note that injection‐related side effects such as redness or pain may—within the frame of this study's applied methodology—be under‐reported, given that only 69.2% of patients completed the patient diaries. This incomplete data collection could result in a recorded frequency of these complications that is lower than the actual occurrence. To strengthen the findings presented herein, incorporating assessments by blinded esthetic physicians could have provided a more objective measure of esthetic outcomes, offering a broader and more well‐rounded perspective on the safety and efficacy of the treatment. Additionally, the study population was drawn from the private sector, which tends to be more costly. This selection bias might affect the generalizability of the findings, as the population may not be representative of the broader demographic, including those who might not have access to private healthcare. Another notable limitation is the gender disparity among the participants, as all subjects were female. This could affect the study's outcomes since men typically have thicker skin with more collagen, which can influence how injectables distribute and settle. The lack of male participants means that the results may not be applicable to men, who might respond differently to PLLA treatments in general and the product used in particular. Lastly, the study's follow‐up period, while substantial, does not capture long‐term effects and satisfaction levels beyond the 150‐day mark, both of which should, however, also be taken into account for a comprehensive understanding of the treatment's efficacy and safety. Future studies should address these limitations by incorporating a more diverse and representative sample, including both genders, and considering objective measures alongside subjective reports to provide a more balanced perspective.

## 6 Conclusion

In this prospective study, we found the collagen biostimulator Rennova Elleva to be safe, well‐tolerated, and associated with high patient satisfaction. Its main ingredient, PLLA, helps achieve long‐lasting results for patients with mild to moderate facial sagging. Patients reported minor injection‐related symptoms, most of which resolved within a few days. Clinically, only one adverse event was noted, with no intervention necessary. Overall, this collagen biostimulator is an effective non‐surgical option for enhancing facial esthetics.

## Author Contributions

Conceptualization: Bruna S. F. Bravo, Thamires Calvacante, and Mariana Calomeni Elias. Methodology, Bruna S. F. Bravo, Thamires Calvacante, and Mariana Calomeni Elias. Statistical analysis: Bruna S. F. Bravo, Maria Carolina Zafra, and Mariana Calomeni Elias. Writing – original draft: Bruna S. F. Bravo and Mariana Calomeni Elias. Writing – review and editing, Thamires Calvacante, Camila Silveira Nobre, Leonardo G. Bravo, and Maria Carolina Zafra. Supervision: Bruna S. F. Bravo and Mariana Calomeni Elias.

## Conflicts of Interest

Bruna S.F. Bravo was hired to develop the study. Thamires Calvacante and Maria Carolina Zafra are medical speakers for Rennova. Mariana Calomeni Elias was hired to perform the study. Leonardo G. Bravo and Camila Silveira Nobre have no conflicts in this study.

## Data Availability

The data that support the findings of this study are available from the corresponding author upon reasonable request.
